# Beyond smoking: Education, marital status and mortality in 513,552 Norwegians

**DOI:** 10.1177/14034948251391598

**Published:** 2026-01-05

**Authors:** Aage Tverdal, Randi Selmer, Dag Steinar Thelle

**Affiliations:** 1 Norwegian Institute of Public Health, Centre for Fertility and Health, Oslo, Norway; 2 Norwegian Institute of Public Health, Department of Mental and Physical Health, Oslo, Norway; 3 Department of Biostatistics, Institute of Basic Medical Sciences, University of Oslo, Norway; 4 School of Public Health and Community Medicine, Institute of Medicine, Sahlgrenska Academy, University of Gothenburg, Göteborg, Sweden

**Keywords:** Education, marital status, smoking, mortality rate

## Abstract

**Background:**

Smoking is closely linked to socioeconomic status and risk of death. We examined the extent to which educational disparities in mortality can be explained by smoking.

**Methods:**

We analysed data from 513,552 men and women aged 25–69 years, excluding individuals with a history of cardiovascular disease, diabetes or cancer. The educational level was lower (less than 10 years), upper (10–12 years) and higher (13 or more years). We employed the direct method to estimate age-adjusted mortality rates and used the Cox proportional hazards model to calculate hazard ratios, using a higher educational level as the reference level. We ran models among non-smokers with interaction terms, educational level and another variable. Based on Akaike information criterion, we performed stratified analyses by marital status among non-smokers.

**Results:**

The hazard ratio for lower educational level in men was 1.38 (1.31–1.45) in non-smokers and 1.57 (1.49–1.66) in smokers. The figures for women were almost identical. Among non-smoking men, the hazard ratio for lower educational level was 1.28 (1.21–1.35) for the ever married and 2.20 (1.93–2.52) for the unmarried men. In women the figures were the same. For non-smoking men and women combined, the population attributable risk ascribed to upper or lower educational level was 17% for the ever married and 35% for the unmarried.

**Conclusions:**

**An educational gradient in mortality persists in the absence of smoking and is more pronounced among the unmarried. Marital status appears to modify the relationship between education and mortality, underscoring the need to consider social and relational factors in health inequality research.**

## Introduction

Smoking has been identified as a contributing factor to various causes of death [1]. Quitting smoking is linked to a notable reduction in mortality, almost on a par with individuals who have never smoked [2]. Higher socioeconomic status, evaluated through diverse metrics such as educational attainment, demonstrates a correlation with lower mortality rates [3]. Marmot uses the term Status syndrome for the social gradient in health [4]. He wrote that the social gradient in health can differ over time and between populations [4]. In Norway, inequalities in mortality were relatively small in a comparison of nine industrialized countries [5,6]. Nevertheless, in Norway, the mortality gap between high and low socioeconomic status has widened despite a decrease in smoking prevalence [7,8]. We studied the educational mortality gradient in smokers and non-smokers, and the educational mortality gradient in non-smokers by marital status.

## Methods

We studied all-cause mortality in 513,552 men and women aged 25–69 years and without a history of cardiovascular disease, diabetes or cancer, by smoking status and educational level. The number of deaths was 54,464. The educational level was lower (less than 10 years), upper (10–12 years) and higher (13 or more years), with prevalences of 20%, 57% and 23%. The data were from the age-40 programme, 1985–1999 and the Cohort of Norway (1994–2003) [9,10].

The participants accrued person-years from date of attendance, once, to date of death or 31 December 2018. Mean person-years was 24 years (range 0.002–31 years). Absolute mortality rates were calculated as deaths per person-year, with age adjustment performed using the direct method based on an internal standard population aged 20–24 to 65–69 years. We employed the Cox proportional hazards model, with person-years as the time variable, to adjust potential confounders including sex, age at examination, total cholesterol, triglycerides, height, body mass index (BMI), systolic blood pressure, physical activity, educational level and unmarried status. Higher educational level was the reference group in the Cox models. We tested deviations from multiplicative joint effect (interaction) by adding a product term in the Cox models, with both terms as continuous variables. The proportional hazards assumption was tested by the scaled Schoenfeld residuals. Among non-smokers, we compared the models with the products of educational level and the remaining independent variables in the Cox models by the Akaike Information Criterion (AIC) and did a stratified analysis by unmarried status, the variable with the minimum AIC. Unmarried status was yes, no, where yes was the unmarried and no was the ever married. We generated a forest plot stratified by marital status and educational level, using estimated coefficients from a Cox proportional hazards model, synthesized through a fixed-effect meta-analysis. We estimated the population attributable risk (mortality) (PAR) of lower or upper educational level as the mortality in the population minus the mortality in the higher educational level in percentage of the first one. There were 497,523 men and women, and 52,922 deaths in the Cox analyses owing to missing information in some covariates.

Height, weight and systolic blood pressure were measured at the screening site. A blood sample was drawn and serum analysed for total cholesterol and triglycerides. Two questions were used to gauge physical activity. One focused on leisure-time activity with alternatives: sedentary, moderate, intermediate, and intensive. The other addressed hard physical activity per week with options ranging from none, less than 1 h, 1–2 h and 3 h or more of hard physical activity per week. For analysis, we defined activity as at least moderate or at least 1 h per week. Other variables were obtained through a questionnaire.

## Results

The mortality increased with decreasing educational level in both smokers and non-smokers (Table I). The hazard ratios comparing lower versus higher educational levels were greater among smokers than non-smokers, for both men and women. The ratio between these hazard ratios was close to 1.13 in both sexes. The mortality difference between these two educational groups was higher among the smokers. The proportional hazards assumption for educational level was not violated for women and smoking men. For non-smoking men, the educational mortality gradient was weaker during the first half of follow-up and stronger in the second half.

**Table I.** Number of deaths, mortality rates and hazard ratios with 95% confidence intervals, by educational level.

Education	Deaths	Rate^a^	Hazard ratio^b^	95% CI	Deaths	Rate^a^	Hazard ratio^b^	95% CI
	Non-smokers				Smokers			
	Men							
Higher	3087	315	1	Ref.	1748	547	1	Ref.
Upper	7025	393	1.18	1.13–1.23	8415	714	1.32	1.25–1.39
Lower	3871	525	1.38	1.32–1.45	5589	915	1.57	1.49–1.66
Per 1 unit			1.18	1.15–1.21			1.23	1.20–1.26
	Women							
Higher	1893	250	1	Ref.	886	353	1	Ref.
Upper	5583	294	1.16	1.10–1.22	5904	447	1.22	1.14–1.31
Lower	4432	395	1.38	1.30–1.46	5073	614	1.55	1.44–1.67
Per 1 unit			1.18	1.14–1.21			1.25	1.22–1.29

a Per 100,000 person-years, adjusted for age.

b Adjusted for age, total cholesterol, triglycerides, body mass index, systolic blood pressure, physical activity and marital status.

CI: confidence interval; Ref.: reference

The hazard ratio for the lower educational level group was smaller for the ever married than for the unmarried (Table II). The same applies to the mortality difference between the two extreme educational groups. Furthermore, the mortality rates were higher among the unmarried than among the ever married.

**Table II.** Number of deaths, mortality rates and hazard ratios with 95% confidence intervals among non-smokers, by marital status and educational level.

Marital status	Education	Deaths	Rate	Hazard ratio	95% CI
	Men				
Ever married	Higher	2770	310	1	Ref.
Ever married	Moderate	5819	368	1.13	1.08–1.18
Ever married	Lower	2913	443	1.28	1.21–1.35
Per 1 unit				1.13	1.10–1.16
Unmarried	Higher	317	370	1	Ref.
Unmarried	Moderate	1206	570	1.61	1.42–1.83
Unmarried	Lower	958	904	2.20	1.93–2.52
Per 1 unit				1.46	1.37–1.55
	Women				
Ever married	Higher	1646	241	1	Ref.
Ever married	Moderate	5031	285	1.12	1.06–1.18
Ever married	Lower	3933	351	1.29	1.21–1.38
Per 1 unit				1.14	1.11–1.18
Unmarried	Higher	247	326	1	Ref.
Unmarried	Moderate	552	424	1.41	1.21–1.64
Unmarried	Lower	499	928	2.20	1.86–2.61
Per 1 unit				1.49	1.37–1.62

a Per 100,000 person-years, adjusted for age.

b Adjusted for age, total cholesterol, triglycerides, body mass index, systolic blood pressure, physical activity and marital status.

CI: confidence interval; Ref.: reference

Figure 1 shows the hazard ratios by marital status and educational level. The educational gradient is more pronounced among unmarried individuals. The PAR for ever married was 17% and for the unmarried 35%.

**Figure 1.**
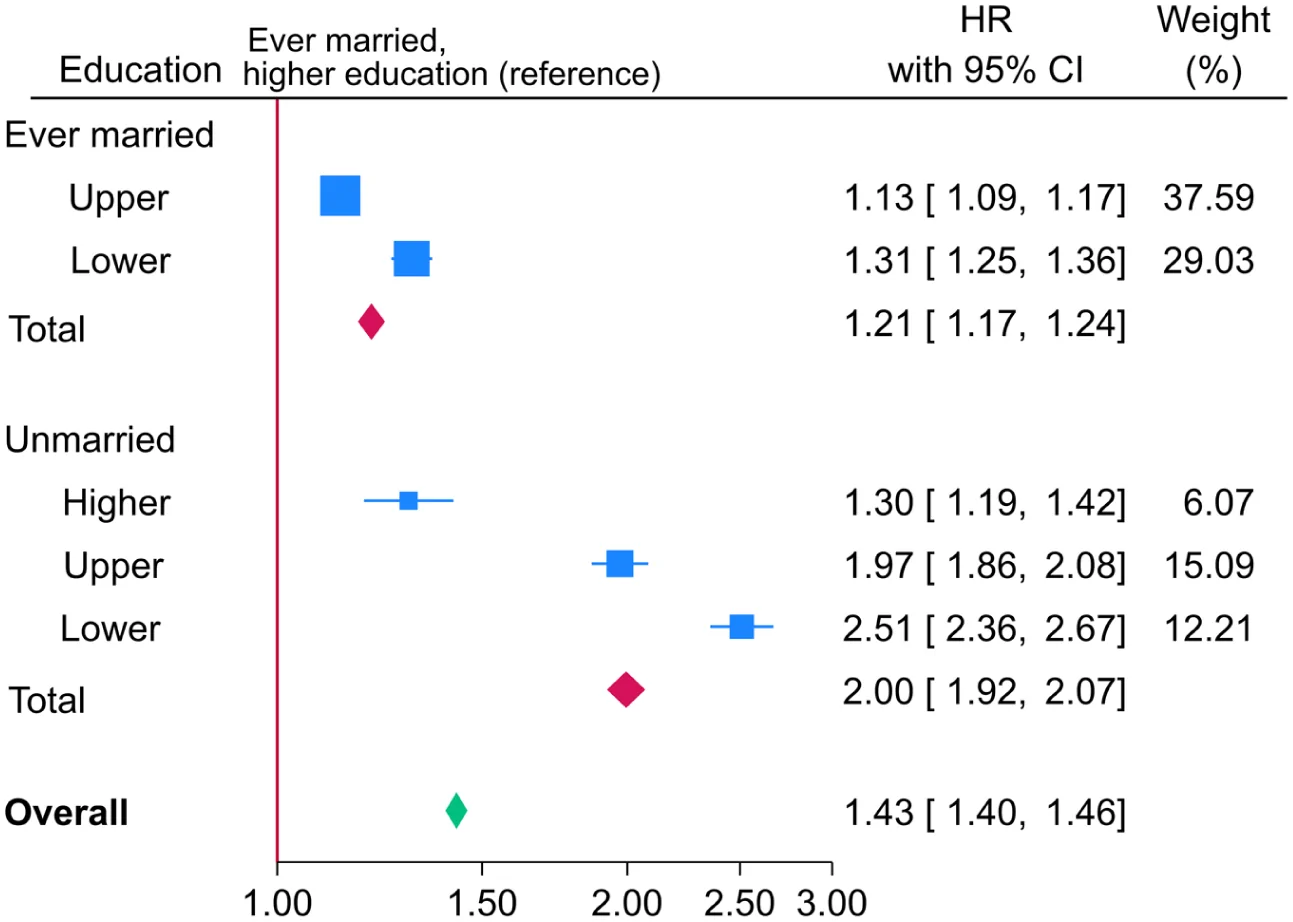
Hazard ratios with 95% confidence intervals, adjusted for age, total cholesterol, triglycerides, body mass index, systolic blood pressure and physical activity, stratified by marital status and educational level. The figure is generated by a forest plot, using estimated coefficients from a Cox proportional hazards model, synthesized through a fixed-effect meta-analysis. CI: confidence interval

## Discussion

This large cohort study confirms that the educational differences in mortality gradient persisted even among non-smokers. Importantly, marital status appears to modify this association: the gradient was considerably stronger among the unmarried.

During the follow-up period, smoking habits and marital status might have changed. While smoking prevalence declined nationally, misclassification is unlikely to fully account for the findings. Changes in marital status, including cohabitation, were not captured. It remains uncertain whether these changes were consistent across educational levels, which might have led to an underestimation of the impact of relationship status.

Some confounders, such as BMI, did change over time, but these changes were consistent across educational groups [11], and adjusting for multiple confounders – including physical activity, BMI and blood pressure – did not eliminate the observed gradient.

There may also be a selection bias linked to unmarried status, for instance ever married individuals tended to be taller, and ever married men had slightly lower education, while the opposite was true for ever married women (data not shown).

Two studies from Norway have documented a growing disparity in mortality rates between socioeconomic groups [7,8]. One explanation is the increasing proportion of unmarried individuals, as this study highlights unmarried status as a significant contributor to educational disparities in mortality. Additionally, the mortality gap between unmarried and married individuals appears to widen with the length of marriage [12], further amplifying the influence of marital status on health outcomes.

In Norway, the proportion of unmarried individuals has increased markedly, from 44% in 1985 to 54% in 2023. During the same period, the prevalence of daily and occasional smokers has decreased from 46% to 16%. These demographic and behavioural changes suggest that educational disparities in mortality now impact a growing share of the population [13,14].

Smoking habits and other risk factors in this study do not fully explain the differences in mortality across educational levels. To better understand the drivers of the educational mortality gradient we must look beyond conventional risk factors.

A limitation of our study is that we have not accounted for alcohol and nutritional intake. The strength of our study is size and a complete follow-up concerning deaths.

In conclusion, an educational gradient in mortality remains evident even after accounting for smoking, with a notably stronger effect among unmarried individuals. Marital status appears to modify the association between education and mortality, underscoring the importance of examining factors beyond cardiovascular risk factors in health inequality research.

## Data Availability

A researcher can apply for access to data at: Access to data – NIPH.

## References

[1] Doll R Peto R Boreham J , et al. Mortality in relation to smoking: 50 years’ observations on male British doctors. BMJ 2004;328:1519. 10.1136/bmj.38142.554479.AE PMC437139 15213107

[2] Tverdal A Selmer R Thelle DS. Smoking history and all-cause, ischaemic heart disease and lung cancer mortality: Follow-up study of 358 551 men and women aged 40–43 years. Tob Control 2025;34:147-53. 10.1136/tc-2023-057977 37963773

[3] McFadden E Luben R Wareham N , et al. Occupational social class, educational level, smoking and body mass index, and cause-specific mortality in men and women: A prospective study in the European Prospective Investigation of Cancer and Nutrition in Norfolk (EPIC-Norfolk) cohort. Eur J Epidemiol 2008;23:511-22. 10.1007/s10654-008-9267-x 18553139

[4] Marmot M . Status syndrome. Significance 2004;1:150-4.

[5] Kunst AE Mackenback JP. The size of mortality differences associated with educational level in nine industrialized countries. Am J Public Health 1994;84:932-7. 10.2105/ajph.84.6.932 PMC1614960 8203689

[6] Mackenbach JP Kunst AE Cavelaers AEJM , et al. Socioeconomic inequalities in morbidity and mortality in western Europe. Lancet 1997;349:1655-9. 10.1016/s0140-6736(96)07226-1 9186383

[7] Strand BH Grøholt E-K Steingrímsdóttir ÓA , et al. Educational inequalities in mortality over four decades in Norway: Prospective study of middle aged men and women followed for cause specific mortality, 1960–2000. BMJ 2010,340:c654. 10.1136/bmj.c654 PMC2827714 20179132

[8] Kravdal Ø . Large and growing social inequality in mortality in Norway: The combined importance of marital status and own and spouse’s education. Popul Dev Rev 2017;43:645-65.

[9] Bjartveit K Wøien G. Cardiovascular disease risk factors in Norway. National Health Screening Service, Oslo 1997, https://www.nb.no/items/URN:NBN:no-nb_digibok_2011103108107 (1997, accessed 30 September 2025).

[10] Næss Ø Søgaard AJ Arnesen E , et al. Cohort profile: Cohort of Norway (CONOR). Int J Epidemiol 2008;37:481-5. 10.1093/ije/dym217 PMC2409050 17984119

[11] Tverdal A. Forekomsten av fedme i to perioder. Tidsskr Nor Lægeforen 2001;121:667-72. 11293345

[12] Gjestrum I . Betydning av endringer i ekteskapelig status for dødelighet i Norge 1975–2002, https://www.duo.uio.no/bitstream/handle/10852/16949/Masteroppgave-Ida-Gjestrum.pdf?sequence=3&isAllowed=y (2010, accessed 30 September 2025).

[13] Statistics Norway. https://www.ssb.no/statbank/table/05813/ (2025, accessed 30 September 2025).

[14] Statistics Norway. 05307: Dagligrøykere og av-og-til-røykere, etter kjønn og alder (prosent) 1973–2023. Statistikkbanken (ssb.no)(2025, accessed 30 September 2025).

